# Implementation of Case Management in emergency departments: the view of the involved staff

**DOI:** 10.1093/eurpub/ckac130.009

**Published:** 2022-10-25

**Authors:** E Schmutz, M Graells, J Moullin, M Kasztura, O Chastonay, M Canepa Allen, O Hugli, JB Daeppen, V Grazioli, P Bodenmann

**Affiliations:** 1 Department of Vulnerabilities and Social Medicine, University of Lausanne, Lausanne, Switzerland; 2 Faculty Health Sciences, School of Pharmacy and Biomedical Sciences, Curtin University, Perth, Australia; 3 Emergency Department, Lausanne University Hospital, Lausanne, Switzerland; 4 Addiction Medicine, Department of Psychiatry, University of Lausanne, Lausanne, Switzerland

## Abstract

**Introduction:**

Frequent users of emergency departments (FUED; ≥ 5 ED visits in the previous 12 months) often present with somatic, psychological and substance use problems. Providing a Case Management (CM) intervention may reduce their number ED visits and improve their quality of life. However, there is limited knowledge about the implementation process of CM.

**Methods:**

This study aimed to introduce CM into the EDs in the French-speaking part of Switzerland and to identify the facilitators, barriers and needs encountered in this process. Semi-structured interviews were conducted with ED involved staff. An inductive content analysis was conducted.

**Results:**

Among 13 invited hospitals, 8 implemented CM (62%); 23 ED staff were sampled from all participating ED: 17 nurses (74%), 5 physicians (22%) and 1 healthcare manager (4%). The average age was 48,48 years (SD = 8,64) and 74% were female. Four main facilitators emerged from the analysis: 1) Direct hierarchy support and flexibility (e.g. time management, supplemental paid hours); 2) Exchange with colleagues (e.g. debriefing, support); 3) Supervision by the research team (training and toolkit consisting of a binder and USB stick containing the study presentation and implementation procedures); and 4) Motivation (pleasure to work on an innovative project, benefit for patients and caregivers). Lack of resources was an unanimously mentioned barrier (e. g., time to identify and contact FUED medical and social support). Finally, participants identified the following needs to enable CM implementation: official and protected time for the project, a dedicated room for CM, at least two team members involved in the project since its initiation with complementary skills (e.g.: somatic, psychiatric and social).

**Conclusions:**

Our study suggests that successful CM implementation is a complex process that, in addition to motivated ED staff, requires significant dedicated resources, such as protected time and a devoted support team.

**Key messages:**

- Future research should establish a case management intervention with resources (time, space, teams with complementary skills) specifically dedicated to this process.
- In order to increase the quality of care, institutions should dedicate more funding for the implementation and sustainability of case management to enable its optimal application by caregivers.

